# The spectrum of paroxysmal nocturnal hemoglobinuria clinical presentation in a Brazilian single referral center

**DOI:** 10.1007/s00277-022-04797-9

**Published:** 2022-02-18

**Authors:** Bruno G. P. Pires da Silva, Natasha P. Fonseca, Luis Fernando B. Catto, Gabriel C. Pereira, Rodrigo T. Calado

**Affiliations:** Department of Medical Imaging, Hematology, and Clinical Oncology, Ribeirão Preto School of Medicine, University of São Paulo, Av. Bandeirantes, 3900, Sala 743, 7º andar – HCRP, Ribeirão Preto, SP 14049-900 Brazil

**Keywords:** Paroxysmal nocturnal hemoglobinuria, Bone marrow failure, Aplastic anemia, Thrombosis

## Abstract

Paroxysmal nocturnal hemoglobinuria (PNH) is a rare hematological disorder caused by the expansion of a hematopoietic clone harboring a somatic genetic variant in the *PIG-A* gene translating into a wide spectrum of clinical and laboratory changes, from intravascular hemolysis, thrombosis, and bone marrow failure to subclinical presentation. In this study, we retrospectively analyzed 87 consecutive cases (39 women; median follow-up, 18 months; range, 0–151 months) in whom a PNH clone was detected by flow cytometry between 2006 and 2019 seen at a single Brazilian referral center. The median age at diagnosis was 29 years (range, 8 to 83 years); 29 patients (33%) were initially classified as PNH/bone marrow failure, 13 (15%) as classic PNH, and 45 (52%) as subclinical PNH. The median overall survival (OS) of the entire cohort was not reached during follow-up, without significant differences between groups. At diagnosis, the median PNH clone size was 2.8% (range, 0 to 65%) in erythrocytes and 5.4% (range, 0 to 80%) in neutrophils. Fourteen patients experienced clone expansion during follow-up; in other 14 patients the clone disappeared, and in 18 patients it remained stable throughout the follow-up. A subclinical PNH clone was detected in three telomeropathy patients at diagnosis, but it was persistent and confirmed by DNA sequencing in only one case. In conclusion, PNH presentation was variable, and most patients had subclinical disease or associated with marrow failure and did not require specific anticomplement therapy. Clone size was stable or even disappeared in most cases.

## Introduction

Paroxysmal nocturnal hemoglobinuria (PNH) is a rare acquired hematopoietic stem cell disorder that manifests in a wide spectrum of clinical signs and symptoms (intravascular hemolysis, thrombosis, bone marrow failure, and evolution to myelodysplastic syndromes and acute leukemia) [1]. It is characterized by the expansion of hematopoietic cell clones harboring acquired somatic pathogenic variants in the *PIGA* gene, located on the X chromosome [2]. Affected cells are deficient in the expression of proteins anchored to the cell membrane via glycosylphosphatidylinositol (GPI), such as CD55 and CD59, two complement regulatory proteins, and consequently are susceptible to complement-mediated cell lysis [3]. The loss of CD55, which inhibits C3 convertase, and CD59, which prevents binding of the membrane attack complex (MAC), causes intravascular hemolysis due to non-regulated complement activation on the erythrocyte membrane [4, 5].

The mechanisms by which clonal expansion occurs is still uncertain, but there are two major hypotheses based on either extrinsic or intrinsic factors [1]. On the one hand, the intrinsic factor theory proposes that the somatic *PIGA* gene variant would increase the hematopoietic stem cell (HSC) proliferation capacity as compared to the non-mutated cells [6]. On the other hand, the extrinsic selection hypothesis proposes that HSCs harboring a *PIGA* mutation would be better fit in a marrow environment dominated by a T cell immune attack, as observed in aplastic anemia (AA) [7, 8]. The second hypothesis provides explanation to the overlap between these two rare disorders (AA and PNH) demonstrated by the common identification of PNH clones in patients with immune aplastic anemia.

PNH diagnosis is confirmed by flow cytometry by the demonstration of GPI-negative cells in at least two cell lineages within white blood cells (WBCs; neutrophils, and monocytes) and red blood cells (RBCs) [9–11]. The PNH clone should be investigated in both cell types (RBCs and WBCs) and the clone size should be determined [3]. For clinical purposes, a PNH clone is established for a given cell lineage when > 1% are deficient for GPI-anchored proteins. The presence of minute clones may be observed, but its significance is uncertain [12–15].

PNH also may be classified based on its clinical presentation [16]: (1) classic PNH, in which clones > 50% are found [17] associated with intravascular hemolysis, anemia, fatigue, hemoglobinuria, smooth muscle dystonia (dysphagia, erectile dysfunction, and abdominal pain), and thrombosis in unusual sites; (2) PNH in the setting of another bone marrow disorder, commonly presenting with cytopenias associated with intravascular hemolysis or thrombosis and a history of aplastic anemia, myelodysplastic syndrome, or other defined myelopathy (e.g., myeloproliferative diseases), with variable clinical presentation according to the underlying condition (PNH/BMF) [3]; and (3) subclinical PNH (PNH-sc), mostly associated with small PNH clones, no clinical evidence of intravascular hemolysis, and uncertain clinical repercussions. This category is commonly observed in association with bone marrow failure. It is important to note that the presence of a GPI-negative clone in patients with bone marrow failure is frequent but is not enough to classify those cases as PNH/BMF. To fit into this category, patients must have a PNH clone with a hemolytic profile and a history of a defined bone marrow disorder. The remaining cases, in which small clonal populations are found, without hemolysis or thrombosis, even in patients previously diagnosed with aplastic anemia, should be classified as subclinical PNH [16].

The treatment of PNH varies according to the clinical presentation. Classic PNH had the greatest impact on its treatment after the introduction of eculizumab, a monoclonal antibody that prevents the formation of the membrane attack complex by blocking C5 cleavage in C5a and C5b [17] and decreases hemolysis and thromboembolic complications and improves survival[1]. However, it has no impact on the underlying stem cell abnormality or on the bone marrow failure [5]. In PNH associated with bone marrow failure syndromes, the treatment is focused on the underlying aplastic anemia, which may involve immunosuppression and/or allogeneic hematopoietic stem cell transplantation [18–20]. In the presence of significant hemolysis or thrombotic events, complement inhibition is warranted [21, 22]. Subclinical PNH, in general, does not require specific treatment, but sporadic monitoring is necessary [20, 23].

The presentation and natural history of PNH cases in Brazil is not well known [24]. In this study, we describe a single-center experience that is a reference center for bone marrow failure syndromes and coagulopathies, in a retrospective series of 87 consecutive patients from 2006 to 2019. We investigated the characteristics of patients with PNH, the treatment modalities available, and the behavior and dynamics of the PNH clone throughout follow-up.

## Patients and methods

### Patient selection

Data were collected from 87 consecutive patients with a GPI-negative clone, through medical record review, seen at the Hospital das Clínicas da Faculdade de Medicina de Ribeirão Preto da Universidade de São Paulo (HCFMRP-USP), between the years 2006 and 2019. The test for PNH clone in red cells and neutrophils (CD55 and CD59 negative) was performed by flow cytometry, and the selection of patients occurred from the moment of the first positive test. As inclusion criteria, we considered those patients who had a PNH clone quantification in erythrocytes or neutrophils higher than 2% in at least one cell type or higher than 1% in both cell lineages. The entire data collection and analysis of the present study were carried out after approval by the Research Ethics Committee (CEP), HCFMRP-USP, and obtained written informed consent from living patients.

We collected information on disease subtype (classic PNH, subclinical PNH, and PNH associated with bone marrow disorder), according to the classification proposed by Parker et al. [16], the clone size in erythrocytes and neutrophils, complete blood counts, LDH quantification (local reference range, 230–460 U/L), thrombosis, therapy modalities, and survival. Data were collected at diagnosis and at the time of the last registered follow-up. We considered as the last follow-up the patient’s last formal hematology appointment at the hospital, the date of death, or the date of the last PNH clone quantification.

Flow cytometric analysis for the PNH clone size was performed using a FACS (fluorescence-activated cell sorter) Canto II Analyzer or Calibur. Until 2019, the PNH clone size of erythrocytes and granulocytes was performed by staining the cells with CD55-PE and CD59-PE. After 2019, the PNH clone was assessed using the following fluorochromes: FLAER-FITC, CD235a-FITC, CD157-PE, CD59-PE, CD15-PERCP-Cy5.5, CD45-APCH7, and CD64-BV510.

### Statistical analysis

Statistical analysis was performed using GraphPad prism 5 program (GraphPad Software, Inc., San Diego, CA, USA). Median was used for descriptive statistics. Overall survival was measured in months from the time of diagnosis to death from any cause and curves generated based on the Kaplan–Meier estimator. Correlation between clone sizes was analyzed using the Spearman’s rank-order correlation. Significance level was set at *α* < 0.05.

## Results

### Patient characteristics

From April 2006 to December 2019, 87 patients met the inclusion criteria for the study. Thirty-nine were women. The median age at diagnosis was 29 years (range, 8 to 83 years); 29 patients (33%) were classified as PNH/bone marrow failure (PNH/BMF; 15 women) with a median age at diagnosis of 26 years (13 to 64 years); 13 (15%) were classified as classic PNH (6 women) with median age at diagnosis of 35 years (range, 20 to 63 years); and 45 (52%) were classified as subclinical PNH (PNH-sc; 18 women) with median age at diagnosis of 29 years (8 to 83 years) (Table 1). At our service, all patients with aplastic anemia are routinely screened for PNH at diagnosis and yearly. In the classic PNH group, all patients were screened for the investigation of intravascular hemolysis, and one also presented an acute thrombotic event at diagnosis. In the PNH-sc group, screening was performed for the investigation of cytopenias (ten patients, one of them ultimately diagnosed with acute panmyelosis with myelofibrosis), thrombosis (two patients), and in thirty-three patients, as routine for aplastic anemia diagnostic screening. In one case, the reason for PNH screening was unclear.

**Table 1:** Demographic characteristics

	Total	PNH/bone marrow failure	Classic PNH	Subclinical PNH
Male	48	14	7	27
Female	39	15	6	18
Age at diagnosis (median and range—years)	29 (8–83)	26 (13–64)	35 (20–63)	29 (8–83)

**Fig. 1:**
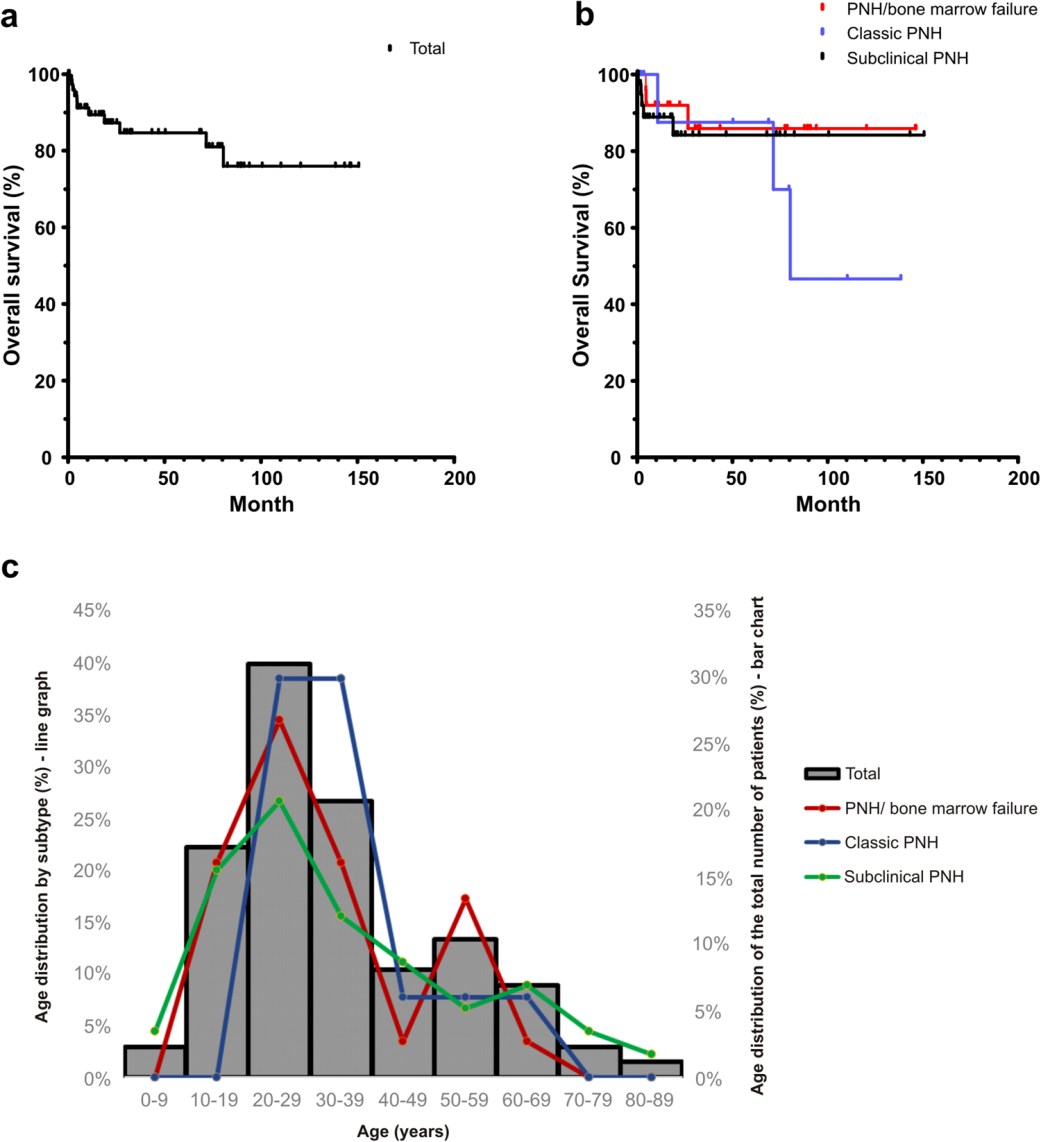
Global and specific subtype overall survival. **a** Analysis of the total cohort overall survival (median not reached) and **b** survival analysis of each subtype: PNH/AA (red), classic PNH (blue), and subclinical PNH (black). **c** Age at diagnosis in the total cohort and in each subtype. The bar chart represents the moment of diagnosis in the total cohort of patients. There’s a peak incidence of classic PNH form (blue line) between the age of 20–29 years and 30–39 years. In the PNH/AA (red line), there is a biphasic distribution with an early peak at 20–29 years and a later peak at 50–59 years. In the subclinical PNH form (green line), the distribution was relatively stable, with no peak incidence being observed, but with a predominance of diagnosis in younger patients at 10–39 years old

The median follow-up was 18 months (0 to 151 months), and the median overall survival (OS) for the entire cohort was not reached during the appraisal (Fig. 1a). When OS was analyzed based on the clinical classification, it was not reached for PNH/BMF and PNH-sc, but it was 80.4 months for classic PNH (Fig. 1b). However, OS was not statistically different among subgroups (*P* = 0.52).

In the subgroup analysis, we observed variation in the age of diagnosis, according to disease presentation, with a peak incidence of classic PNH between 20 and 39 years. In the PNH/BMF group, we observed a bimodal distribution with an early peak at 20–29 years and a later peak at 50–59 years. In the PNH-sc form, the distribution was relatively stable with a peak at 20–29 years (Fig. 1c).

In our cohort, only five patients (6%) had thrombotic events and four had the first thrombotic episode at the time of diagnosis; only one patient had the first thrombotic episode during follow-up. Two were classified at diagnosis as classic PNH, one as PNH/BMF and two as subclinical PNH. In these two cases of subclinical PNH, the thrombotic events were attributed to other risk factors. Both had small clones (less than 5%) with no evidence of any hemolysis on laboratory tests.

**Fig. 2:**
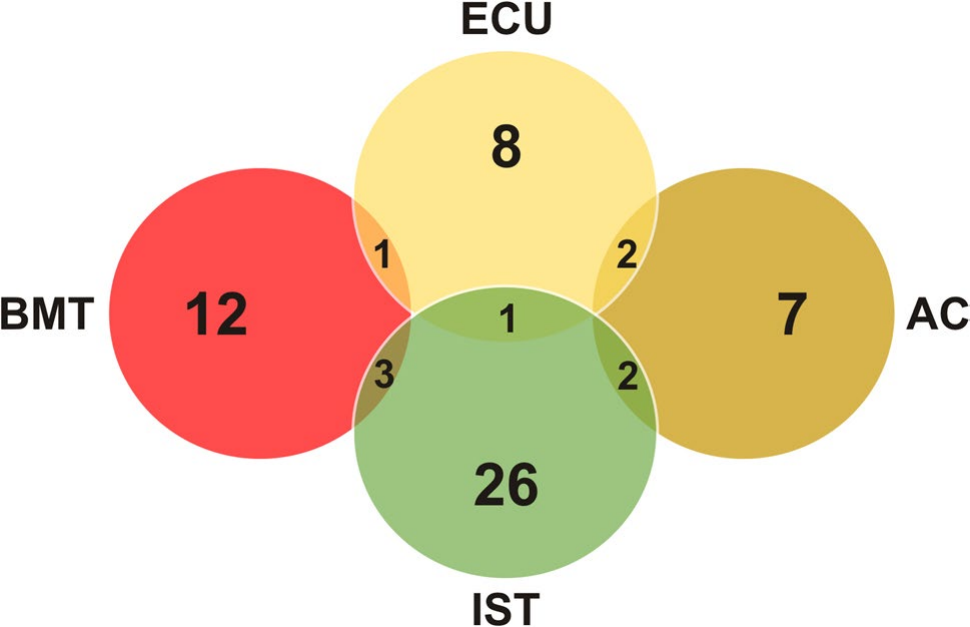
Treatment modalities. ECU, eculizumab; AC, anticoagulation; IST, immunosuppressive therapies; BMT, bone marrow transplantation. The intersections of the diagrams represent patients that were treated with two different modalities. Five out of the eleven patients anticoagulated with warfarin had previous venous thrombotic episodes, and the others received primary prophylaxis. The remaining patients received transfusion support as the main therapy (13 patients), treatment directed to underlying hematologic condition, or clinical observation only (not shown in the figure)

Twelve out of 87 patients were treated with eculizumab, 32 received immunosuppressive therapy, consisting of rabbit antithymocyte globulin (dose of 1 to 3 mg/kg/day for 5 days) and cyclosporine (initial dose, 6 mg/kg/day), with or without the addition of eltrombopag. Twelve patients failed initial immunosuppressive treatment and required additional therapies (more immunosuppression or allogeneic hematopoietic stem cell transplantation). A total of 16 patients (18%) underwent allogeneic bone marrow transplantation during follow-up (as initial or rescue therapy). One patient received infusion of mesenchymal stromal cells [25]. Eleven patients were anticoagulated with warfarin, of which five had previous venous thrombotic episodes, and in the others as primary prophylaxis. The other patients received transfusion support as the main therapy (13 patients), treatment directed to underlying hematologic conditions, or clinical observation only (Fig. 2).

### PNH clone dynamics

To elucidate the PNH clone dynamics over time in this cohort, we determined the clone size in red cells and neutrophils at diagnosis and last follow-up (median, 18 months; range, 0 to 151 months) and classified evolution profiles as proposed by Schrezenmeier et al. (“expansion,” “disappearance,” or “persistent”). “Expansion” was considered when a > 10% increase in clone size was observed [26, 27].

At diagnosis, the median PNH clone size in erythrocytes was 2.8% (range, 0 to 65%) and 5.4% (0 to 80%) in neutrophils. At the time of the last follow-up, the median size in erythrocytes was 1.9% (0 to 98%) and in neutrophils it was 7.2% (0 to 97%). In the classic PNH group, the median clone size in red cells at diagnosis was 34% (5 to 65%) and 18% (0 to 80%) in neutrophils. At last follow-up, the median size in red cells was 80% (1.5 to 98%) and 83% (3.4 to 97%) in neutrophils. In the PNH/BMF group, the median clone size in erythrocytes at diagnosis was 6% (0 to 32%) and 23% (1 to 68%) in neutrophils. At last follow-up, the median size was 6.4% (0 to 98%) in erythrocytes and 24% (0 to 94%) in neutrophils. In the PNH-sc group, the median size in erythrocytes at diagnosis was 0 (0 to 10%) and 3.2% (0 to 50.68%) in neutrophils. At last follow-up, the median size was 0 (range, 0 to 74.27%) in erythrocytes and 1.5% (0 to 87%) in neutrophils. These results are summarized in Table 2.

**Table 2:** Laboratory findings

	Total	PNH/AA	Classic PNH	Subclinical PNH
Erythrocytes clone (diagnosis) — %	2.84 (0–65)	6 (0–32)	34 (5–65)	0 (0–10)
Erythrocytes clone (last follow-up) — %	1.91 (0–98)	5.36 (0–98)	80.4 (1.51–98)	0 (0–74.27)
Neutrophils clone (diagnosis) — %	5.4 (0–80)	20.5 (1–68)	18 (0–80)	3.22 (0–50.68)
Neutrophils clone (last follow-up) — %	7.21 (0–97)	16.97 (0–94)	82.75 (3.42–97)	1.5 (0–86.53)
Hemoglobin (diagnosis) — g/dL	9.3 (2.1–16.7)	9.1 (2.1–15.7)	9.7 (5.3–14)	9.4 (4.6–16.7)
Neutrophils (diagnosis) — 10^3^/mm^3^	1100 (0–16900)	1100 (200–4700)	2750 (400–5400)	900 (0–16,900)
Platelets (diagnosis) — 10^3^/mm^3^	40,000 (1000–456,000)	32,000 (1000–144,000)	146,000 (33,000–244,000)	37,000 (3000–456,000)
LDH (diagnosis) — UI/L	528 (126–7734)	795 (314–3756)	2667 (485–7734)	389 (126–1095)

**Fig. 3:**
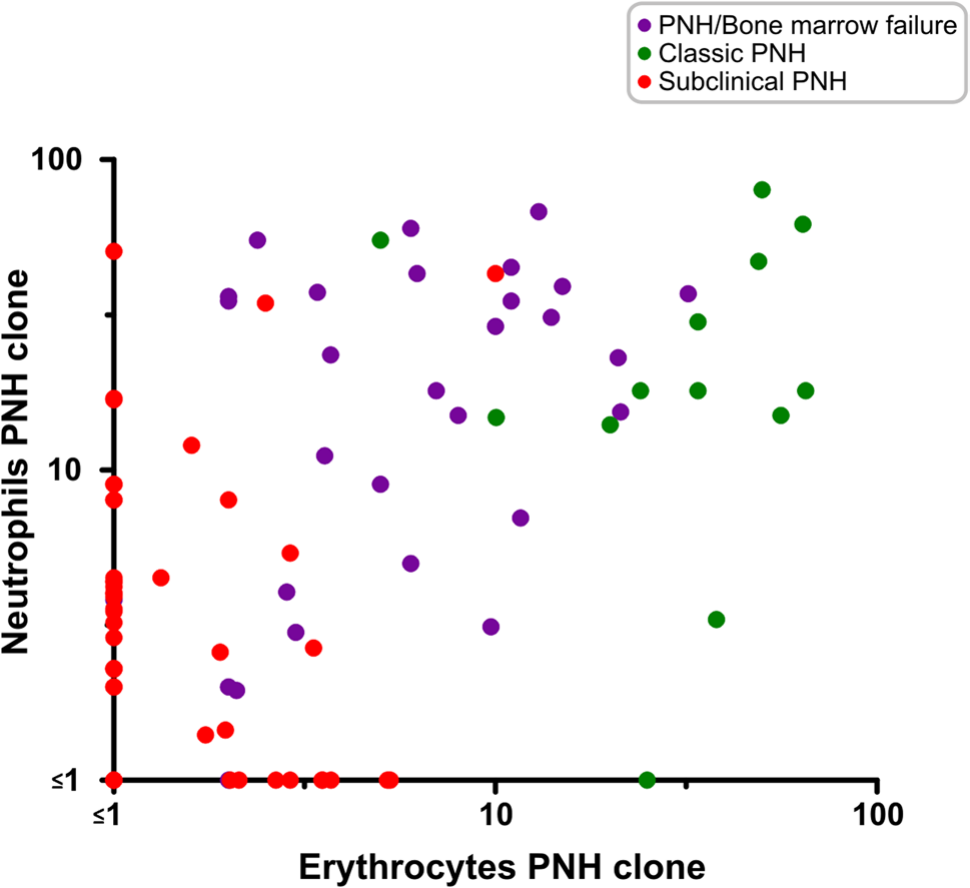
Distribution of the clone size in each disease subtype at diagnosis. Axes in logarithmic scale: purple dots, PNH/bone marrow failure group; green dots, classic PNH group; red dots, subclinical PNH group. Most of the patients showed a direct correlation between clone size in erythrocytes and neutrophils at the moment of first screening (%). In the classic PNH group, the median clone size in red cells at diagnosis was 34% (5 to 65%) and 18% (0 to 80%) in neutrophils. In the PNH/AA group, the median clone size in erythrocytes at diagnosis was 6% (0 to 32%) and 23% (1 to 68%) in neutrophils. In the subclinical PNH group, the median size in erythrocytes at diagnosis was 0 (0 to 10%) and 3.2% (0 to 50.68%) in neutrophils

Most patients showed a positive correlation between clone sizes in erythrocytes and neutrophils at the first measurement (Spearman r, 0.4454; *P* < 0.0001; Fig. 3), as previously described by others [27]. Classic PNH patients showed a significantly larger clone in erythrocytes and neutrophils at diagnosis as compared with subclinical and PNH/BMF. Although PNH-sc patients had significantly smaller clones as compared to patients with classic PNH and PNH/BMF, relatively larger clones (> 10%) in neutrophils were observed in five cases. In these specific patients, no thromboembolic event or sign of hemolysis were documented. All had severe neutropenia at the time of PNH clone screening. Thus, despite the large percentage of GPI-negative neutrophil clones, the absolute number of GPI-negative neutrophils was very low. Furthermore, the red blood cell clones were small, supporting the subclinical classification. One limitation in the long-term clinical analysis of these cases is that two patients died soon after PNH screening, two lost follow-up, and one underwent bone marrow transplant, precluding a comprehensive characterization of the clone dynamics.

**Fig. 4:**
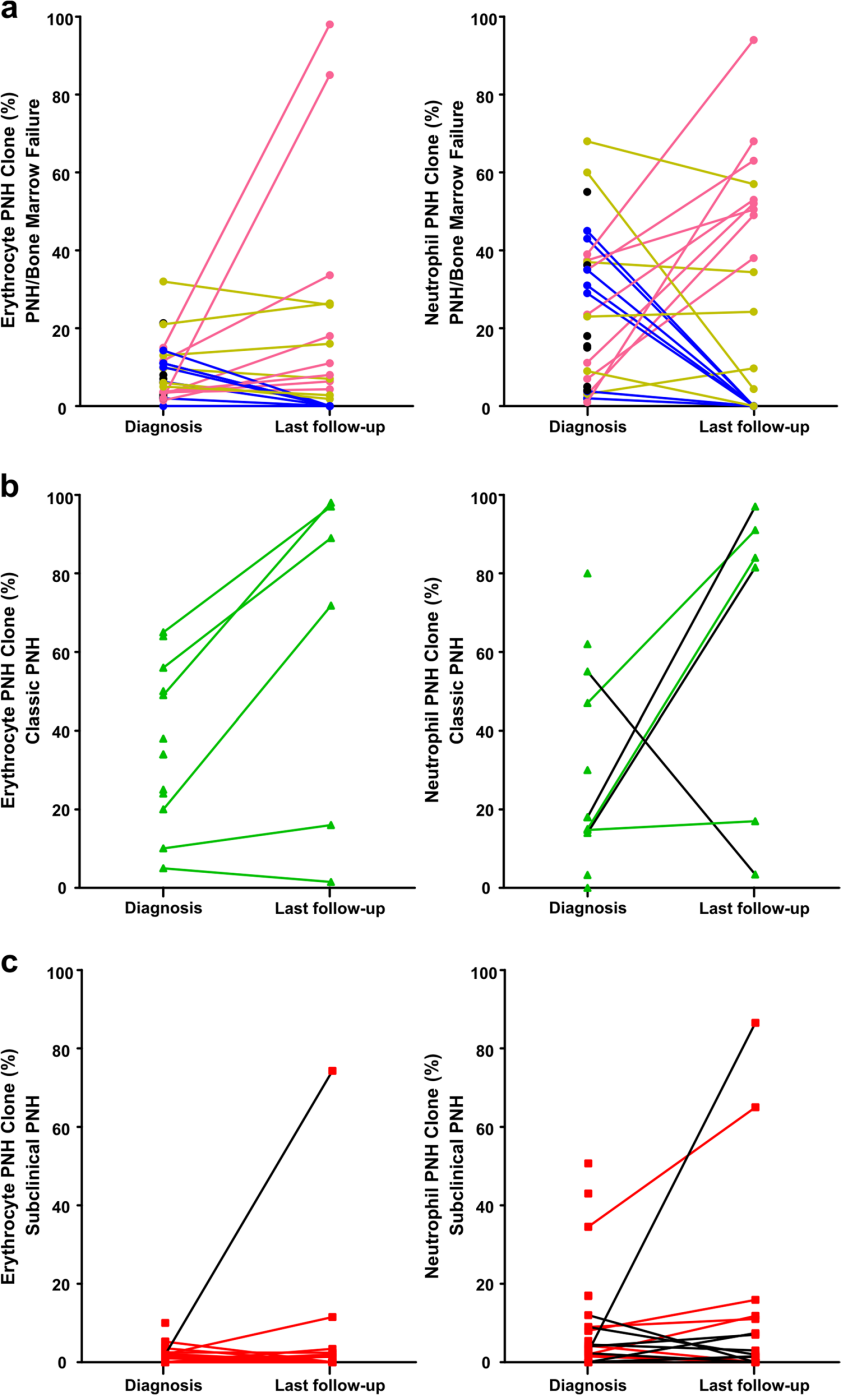
PNH clone dynamics at diagnosis and the last follow-up at PNH/AA failure group (**a**), classic PNH (**b**), and subclinical PNH (**c**). On the PNH/AA group, the expansion in the clone size was represented in pink lines, the disappearance in blue lines, and the stable clones in yellow lines. The lines represent only patients with more than one PNH clone quantification during follow-up (46 patients). In 14 patients (16%), there was an expansion of the clone size (increase > 10% in at least one cell lineage), of which eight were classified as PNH/AA, four as classic PNH, and two as subclinical PNH. The clone disappeared in other 14 patients (16%; seven with PNH/AA and seven with subclinical PNH). Clone disappearance was not observed in any patient with classic PNH. Eighteen patients (21%) showed stability of the clone size through follow-up. The change in the clinical presentation occurred in three patients studied (3.5%). One of them had the PNH/bone marrow failure syndrome and evolved to a subclinical clone. Another patient presented initially as classic PNH evolved to the subclinical form. And last, a patient with a subclinical clone associated with aplastic anemia evolved to an PNH/AA with a hemolytic/thrombotic profile

According to the previously defined criteria for expansion, persistence, and disappearance of the clone [26, 27], we observed that in 14 patients (16%), there was an expansion (increase > 10% in at least one cell lineage), of which eight were classified as PNH/BMF, four as classic PNH, and two as PNH-sc. The clone disappeared in other 14 patients (16%; seven with PNH/BMF and seven with PNH-sc). We did not observe clone disappearance in any patient with classic PNH, although one patient had a significant reduction in clone size in both neutrophils and erythrocytes, evolving to the subclinical form during follow-up. In 18 patients (21%), the clone remained stable through follow-up (six PNH/BMF, two classic PNH, and ten PNH-sc). For 41 patients, there was only one measurement available and clone dynamics was not determined. The curves representing the GPI-negative clone dynamics over follow-up are shown in Fig. 4a (PNH/AA), Fig. 4b (classic PNH), and Fig. 4c (subclinical PNH).

In our cohort, we detected the presence of subclinical PNH clones in three patients diagnosed with short telomeres and marrow failure; one had a heterozygous pathogenic *TERT* gene variant (c.2212C > A p.Asp 718Glu), with a PNH clone size of 4% in neutrophils at diagnosis and < 1% in RBC. The clone remained stable during follow-up (7% in neutrophils in the last measurement). In this patient, a somatic variant in the *PIGA* gene (c.486delC: p.V162fs) was detected with an allele frequency of 1.8% at diagnosis that increased to 17% 2 years later. The patient eventually received a matched-unrelated donor hematopoietic stem cell transplant and the PNH clone disappeared. The second patient had a heterozygous pathogenic *TERT* gene variant (p.Arg865His) with a PNH clone in red cells of 3.53% at diagnosis, and the third patient had a homozygous pathogenic *TERC* gene variant (n.110_113delGACT), with a clone size of 2.13% in red cells at diagnosis. The last two patients had no detectable PNH clones in neutrophils, and both red cell PNH clones disappeared within 1 year of follow-up. No somatic variant in the *PIGA* gene was detected in these two cases.

## Discussion

We report the natural history of 87 consecutive patients with a positive PNH test in a single Brazilian referral center and found that most patients (52%) presented PNH-sc and only 15% had classic PNH. The median survival was not reached during an 18-month median follow-up. In most cases, the clone size was stable or disappeared, especially in subclinical PNH. Clone expansion mainly occurred in classic PNH.

As demonstrated by Scheinberg et al., approximately one third of AA patients have a PNH clone > 1% at diagnosis [7, 8], and at our service, all patients with bone marrow failure are regularly screened for PNH at diagnosis. However, most clones detected in these AA patients showed an indolent clinical course without hemolysis or thrombosis and were classified as PNH-sc and not PNH/BMF. In addition to routine screening in these cases, it is routine at our service to screen for a PNH clone in the investigation of patients with hemolytic anemia, cytopenias, and/or thrombosis. Our service also is a reference center for coagulopathies, but in our series, most cases where a PNH clone was detected were associated with marrow failure and not thrombosis. This epidemiologic profile may still be biased by local characteristics, as observed in other centers [27–29].

We noticed similar numbers of patients in which the clone expanded (14 patients), disappeared (14 patients), or remained stable (18 patients). However, looking at these subgroups individually, we found that in the group where there was clone expansion, 12 out of 14 patients (85%) already had relatively large clones (> 10%) at the moment of diagnosis, initially classified as classic PNH or PNH/BMF, and only two patients were initially classified as PNH-sc. The factors leading to such expansion in these patients are still unclear. Some authors suggest that the acquisition of additional mutations in genes other than *PIGA* or even somatic variants in the *PIGA* gene may confer an additional proliferative advantage for the clones [27, 30, 31]. It is unclear whether the treatment modality may play any role in the clone dynamics, although some studies tried to elucidate this issue after immunosuppression with anti-lymphocyte globulin and cyclosporine [8] and after high doses of cyclophosphamide [32].

We were unable to observe a similar pattern in the group of patients in which the clone disappeared: 6 out of 14 patients (42%) had large clones (> 10%) prior to their disappearance. However, no patient with the classic PNH presentation experienced clone disappearance, regardless of treatment.

The change in the clinical presentation subtype was uncommon in our cohort; it occurred in three out of 87 patients (3.5%). One of them started his clinical presentation as PNH/BMF and evolved to PNH-sc after immunosuppression with rabbit anti-lymphocyte globulin and cyclosporine. Another patient presented initially as classic PNH and evolved to PNH-sc. However, this patient never underwent any specific treatment for his condition. Finally, a patient with a subclinical clone associated with aplastic anemia (PNH-sc) evolved to an PNH/BMF with evident hemolysis after immunosuppression with rabbit anti-lymphocyte globulin and cyclosporine, requiring anticomplement therapy with eculizumab. The factors influencing clone change are not elucidated, and we hypothesize that, in these cases, immunosuppression may have favored the selection subclones with different proliferative capacities [33, 34].

The majority of patients in our cohort were treated with immunosuppression, with or without the addition thrombopoietin receptor (TPO-R) agonist, or bone marrow transplantation. The main indication for treatment was the underlying bone marrow failure and not the PNH clone itself, which associates with the majority of patients with PNH-sc. Twelve out of 87 patients received anticomplement therapy during follow-up. Of these, the majority (eleven patients) had clones with a clinical hemolytic/thrombotic profile (4 with PNH/BMF). Only one patient initially presented as PNH-sc and eventually evolved to classic PNH. Only eleven patients were anticoagulated with warfarin, six of which as primary prophylaxis, in agreement with the low incidence of thrombotic events in our cohort. The remaining patients received transfusion support.

Our analysis identified the presence of a subclinical PNH clone in 3 patients previously diagnosed with short telomeres carrying a pathogenic germline telomerase gene variant and marrow failure; in two patients, the PNH clone disappeared within 1 year of follow-up. We hypothesize that the small clones have been falsely identified in these two cases, due to limitations in the specificity of our flow cytometry method to detect very small clones at the time. Alternatively, somatic mutations occurred in more differentiated hematopoietic precursors, and not in the hematopoietic stem cell, eventually disappearing during follow-up [12, 13, 35, 36]. In one case, however, a PNH clone was detected in 4% of neutrophils and maintained stable over 2 years in a patient with a pathogenic *TERT* variant (D718E) with short telomeres, aplastic anemia, and a family history of idiopathic pulmonary fibrosis (second cousin once removed). A pathogenic variant in the same codon (D718N) had been previously described in a patient with dyskeratosis congenita [37]. Interestingly, a somatic variant in the *PIGA* gene was detected at two different time-points. To the best of our knowledge, this is the first report of a PNH clone in a patient with telomere disease. The pathogenicity of the *TERT* gene variant is strong (short telomeres, family history, previous report) and the PNH clone was detected by flow cytometry and next-generation sequencing in at least two independent samples. The reason for the PNH clone appearance is not clear. One hypothesis is that an immune mechanism may contribute to marrow failure in this case. Alternatively, the PNH clone may be the result of genetic drift [38].

We also identified one patient with acute panmyelosis with myelofibrosis carrying a PNH clone. GPI-negative clones of 1.6% in red cells and 12% in neutrophils were detected at diagnosis but disappeared 1 month later. The patient died from complications of his underlying disease in a few months, and we were unable to quantify the clone in subsequent measurements.

In conclusion, the factors that influence the clinical features of the PNH clone and its dynamics over time are still poorly understood, and the epidemiology and natural history of patients with PNH in Brazil is still scarce. The present study contributes to better understand the characteristics of patients with PNH in our population and the fate of PNH cells, according to possible clinical and laboratory features that may contribute to more aggressive or indolent presentations of the disorder.

## Data Availability

The datasets generated and analyzed during the current study are available from the corresponding author on reasonable request.
